# Antioxidants: The Chemical Complexity Behind a Simple Word

**DOI:** 10.1021/acs.accounts.5c00552

**Published:** 2025-09-10

**Authors:** Annia Galano

**Affiliations:** Departamento de Química, Universidad Autónoma Metropolitana-Iztapalapa, Ave. Ferrocarril San Rafael Atlixco 186, Col. Leyes de Reforma 1A sección, Alcaldía Iztapalapa, 09310 Mexico City, Mexico

## Abstract

What does the word antioxidant mean? Antioxidants are supposed to be nontoxic, versatile molecules capable of counteracting the damaging effects of oxidative stress (OS). Thus, when evaluating a candidate molecule as an antioxidant, several aspects should be considered. Antioxidants are more than free radical scavengers. Other routes may contribute to their protection against OS, including modulation of the redox enzymatic system, preventing free radical formation, and repairing oxidized biomolecules. However, molecules intended as antioxidants can also exhibit pro-oxidant or toxic effects. Thus, understanding the full complexity of their chemistry is crucial for making reliable predictions about their activity.

This Account focuses on computational tools that can assist in addressing such a challenging task. Some key aspects to consider when evaluating the potential antioxidant activity (AOX) of a molecule using these tools are (i) its absorption, distribution, metabolism, and excretion (ADME) properties; (ii) the effects of solvent and pH on its speciation and reactivity; and (iii) the toxicity of the molecule, its metabolites, and the products of the reactions it may undergo *in vivo*. While computational tools offer unique insights into the chemoprotective effects of antioxidants, care must be taken when assessing the data they produce. For example, reactivity descriptors alone are seldom enough to make reliable predictions on AOX. The thermodynamics and kinetics of the reaction pathways contributing to it frequently rule the antioxidant performance. The selected method of calculation should be reliable for the task at hand, since it influences the numerical outcome. Using some references for comparison allows adding context to the calculated data.

We have developed two protocols that can be combined to include those aspects into computational studies of antioxidants: the Quantum Mechanics-Based Test for Overall Free Radical Scavenging Activity (QM-ORSA) and the Computer-Assisted Design of Multifunctional Antioxidants Based on Chemical Properties (CADMA-Chem). Some examples of the application of these protocols are discussed herein. They illustrate the diversity of reaction mechanisms and environmental conditions that modulate AOX, considering potential benefits and risks. These protocols provide a theoretical framework for investigating AOX that allows straightforward comparison with experimental results. They can be applied to known antioxidants for gaining insight into observed behavior as well as in the development of new antioxidants intended as potential drug candidates for the treatment of OS-related diseases.

This work aims to promote comprehensive investigations into antioxidant chemistry, contribute to the interpretation of the results obtained from calculations, and encourage the development of safe, efficacious molecules that ameliorate the harmful effects of OS on human health.

## Graphical abstract

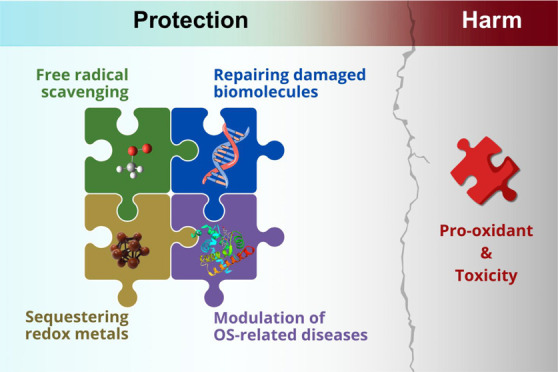

## Key References

-

Galano, A. ; Medina, M. E. ; Tan, D. X. ; Reiter, R. J. Melatonin and its Metabolites as Copper Chelating Agents and their Role in Inhibiting Oxidative Stress: A Physicochemical Analysis. J. Pineal Res. 2015, 58, 107–116 25424557 10.1111/jpi.12196.^1^ *Using the melatonin family as a study case, this paper presents the role of antioxidants as OH inactivating ligands when they are in the presence of redox metals. It illustrates the different chemical routes through which molecules can inhibit Fenton-like reactions, thus preventing the formation of highly damaging free radicals.*
-

Alvarez-Idaboy, J. R. ; Galano, A. On the Chemical Repair of DNA Radicals by Glutathione: Hydrogen vs Electron Transfer. J. Phys. Chem. B 2012, 116, 9316–9325 22799525 10.1021/jp303116n.^2^ *This paper analyzes the different types of damage that can affect biomolecules, in particular DNA, and how antioxidants may be capable of reversing this damage. The role of pH and the importance of using models as realistic as possible in the calculations are discussed.*
-

Castañeda-Arriaga, R. ; Pérez-González, A. ; Reina, M. ; Alvarez-Idaboy, J. R. ; Galano, A. Comprehensive Investigation of the Antioxidant and Pro-oxidant Effects of Phenolic Compounds: A Double-Edged Sword in the Context of Oxidative Stress? J. Phys. Chem. B 2018, 122, 6198–6214 29771524 10.1021/acs.jpcb.8b03500.^3^ *This paper addresses the possibility that phenolic antioxidants can propagate free radical chain reactions and protein arylation. The various reaction mechanisms that may be involved in such undesirable effects are discussed in detail. Both anti- and pro-oxidant behavior are quantitatively compared and analyzed.*
-

Galano, A. ; Alvarez-Idaboy, J. R. A computational methodology for accurate predictions of rate constants in solution: Application to the assessment of primary antioxidant activity. J. Comput. Chem. 2013, 34, 2430–2445 23939817 10.1002/jcc.23409.^4^ *This paper presents a computational protocol for accurately estimating rate constants of radical–molecule reactions in solution, validated against experimental data. It is commonly referred to as the QM-ORSA test and predicts free radical scavenging activity based on thermochemistry and kinetics.*
-

Guzman-Lopez, E. G. ; Reina, M. ; Perez-Gonzalez, A. ; Francisco-Marquez, M. ; Hernandez-Ayala, L. F. ; Castañeda-Arriaga, R. ; Galano, A. CADMA-Chem: A Computational Protocol Based on Chemical Properties Aimed to Design Multifunctional Antioxidants. Int. J. Mol. Sci. 2022, 23, 13246 36362034 10.3390/ijms232113246PMC9658414.^5^ *This paper presents a protocol for the design of versatile antioxidants meant to be used in the treatment of OS-related diseases, including Alzheimer’s and Parkinson’s. It includes considering toxicity, ADME properties, and synthetic accessibility. The importance of simultaneously considering a wide range of chemical behaviors in the design of new antioxidants is discussed.*

## Introduction

The amount of data gathered so far, supporting the risks of oxidative stress (OS) and the benefits of antioxidants to our health, is overwhelming. OS is triggered by oxidant chemicals and pro-oxidant enzymes, and frequently mediated by free radicals, with both exogenous and endogenous factors contributing to it (Figure 1). Multiple investigations have related OS with a variety of diseases, including cancer, neurological disorders, arthritis and diabetes. At the same time, antioxidants have emerged as a practical alternative to counteract the deleterious effects of OS. They have become a focus of scientific research and marketing strategies. Thus, it is not surprising that the word *antioxidant* is now part of our daily language. But how much do we understand about the ways in which they provide such benefits? And how many of the molecules assumed as antioxidants came from enthusiastic interpretation and wishful thinking?

**Figure 1:**
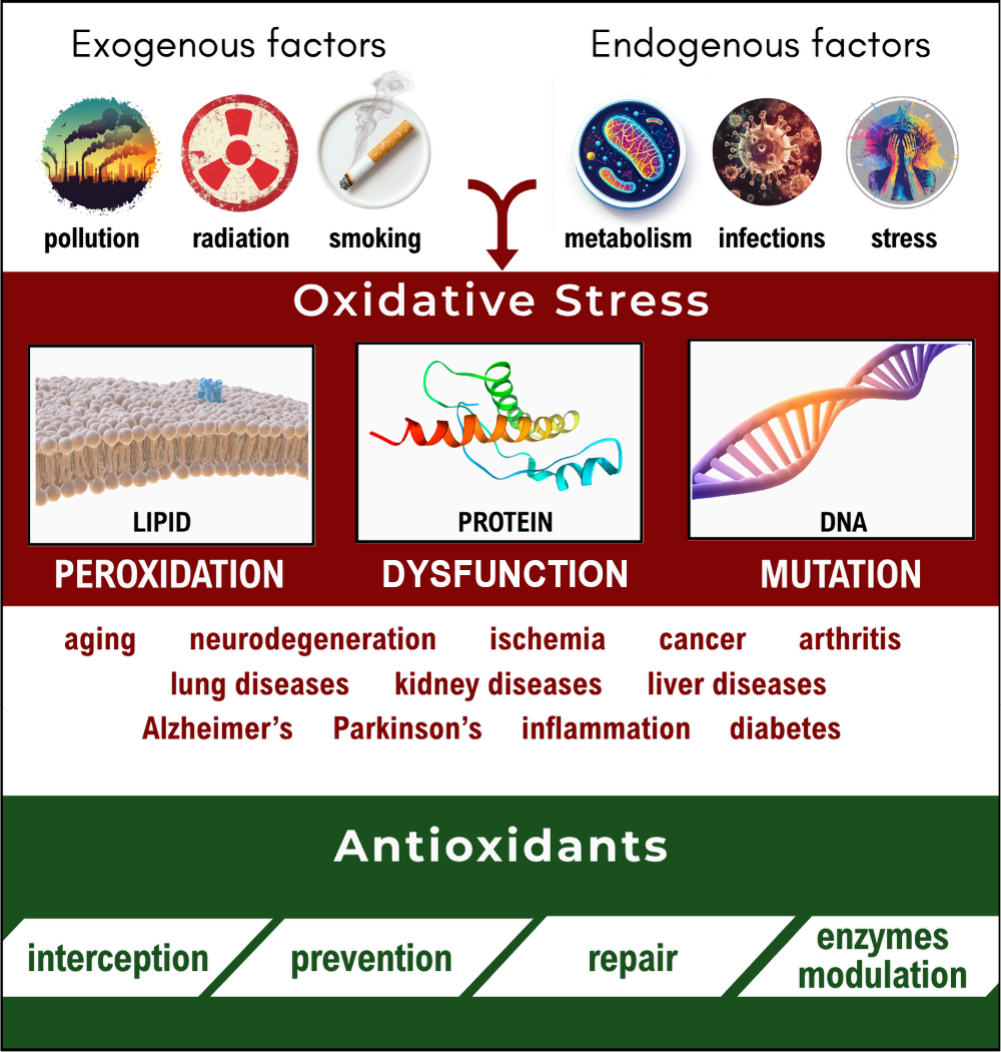
Schematic representation of factors contributing to OS, its consequences, and the role of antioxidants on counteracting it.

Although antioxidants are best known as free radical scavengers (interception or AOX-I, Figure 1), they are much more than that. They can decrease the number of OH radicals endogenously produced by inhibiting Fenton-like processes (prevention or AOX-II); restore biomolecules that are oxidatively damaged to their pristine forms (repairing or AOX-III); and interact with our redox enzymatic system.^6^ On the contrary, they might be toxic or promote molecular damage. Thus, the complex behavior of antioxidants usually requires multiple investigations to confirm a candidate molecule as an efficient antioxidant. Combining experimental (*in vitro* and *in vivo*) and computational (*in silico*) techniques to address such a challenging task is the best approach, albeit frequently difficult to achieve.

This Account provides an overview of computational protocols developed in our group to investigate the chemistry of antioxidants, including those known as the “Quantum Mechanics-Based Test for Overall Free Radical Scavenging Activity” (QM-ORSA)^4^ and the “Computer-Assisted Design of Multifunctional Antioxidants Based on Chemical Properties” (CADMA-Chem).^5^ The discussion includes important aspects to consider when modeling chemical reactions related to antioxidant activity in solution and those that need to be considered for consumption by humans. Some of these aspects are the physicochemical properties related to absorption, distribution, metabolism, and excretion (ADME); toxicity; p*K*_a_ and acid–base equilibria; thermochemistry; kinetics, and interactions with receptors involved in OS-related diseases.

Antioxidants are ubiquitous in nature and can also be built by design. In both cases, understanding the various aspects of their chemistry is crucial to assess the potential health benefits and their risks.

## Scavenging Free Radicals (Interception, AOX-I)

Several reaction mechanisms may contribute to the free radical scavenging activity of antioxidants, also referred to as interception or AOX-I. The most common ones are summarized in Scheme 1, using phenol as a hypothetical antioxidant. Formal hydrogen atom transfer (*f*-HAT) refers to the transfer of a proton and an electron from the donor (the antioxidant) to the acceptor (a radical) as a single entity. The word *formal* is used because such a transfer can take place in at least two different ways, although in both cases the final products are the same: the hydrogen atom can be transferred as a single entity (HAT) or via a proton-coupled electron transfer (PCET) mechanism. Thus, the *f* in the acronym refers to the overall process, without specifying the route involved. More details on the differences between HAT and PCET, and their chemical implications, can be found elsewhere,^7^ as well as comprehensive discussions about PCET^8^ and its variants.^9^

**Scheme 1:**
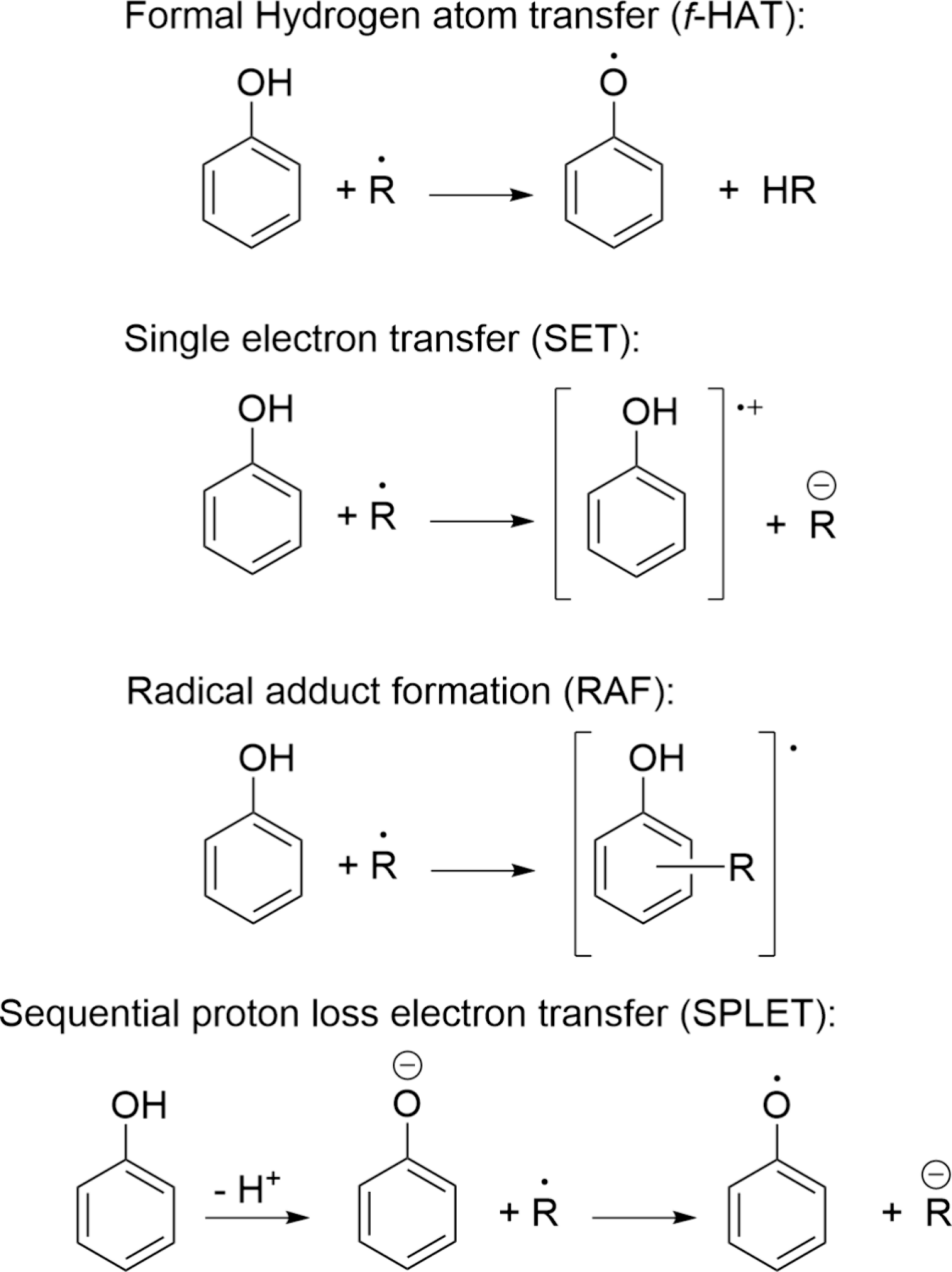
Some of the Most Common Reaction Mechanisms Involved in the Free Radical Scavenging Activity of Antioxidants (AOX-I), Using Phenol to Illustrate Them

Ionization energies (IEs) and bond dissociation energies (BDEs) are among the most used reactivity descriptors for antioxidants since they are directly related to the thermochemistry of single electron transfer (SET) and *f*-HAT processes, respectively.^10^ They are frequently used to anticipate the potential role of candidate antioxidant molecules as electron and H atom donors in AOX-I. It is then desirable that these descriptors are estimated as accurately as possible. Some examples of computational approaches that allow this are the property-specific atom-centered potential approach for BDEs,^11^ and the electron propagator theory for IE.^12^

However, BDEs and IEs are frequently not sufficient to describe AOX-I. The importance of being cautious when predicting antioxidant activity based only on thermochemical descriptors has been previously highlighted.^13^ For example, very low IE values might be misleading since they correspond to Gibbs free energy of SET reactions in the inverted zone of the Marcus parabola, i.e., albeit thermochemically feasible, they would take place at very low rates. Thus, in such cases kinetic calculations are required to assess the contributions of SET processes in AOX-I.^14^ Other reaction mechanisms, like radical adduct formation (RAF), are not reflected by hydrogen BDE and IE values of the candidate molecule, while this route may contribute to AOX-I to a larger extent than *f*-HAT, like it is the case for carotenoids.^15^

QM-ORSA^4^ was designed to explore all the chemical routes that may be involved in AOX-I. It is used to compute the thermochemistry and kinetics of such routes and it generally produces results in excellent agreement with experimental data (Figure 2). It has been successfully used by us^16–21^ and other researchers^22–27^ to investigate AOX-I.

**Figure 2:**
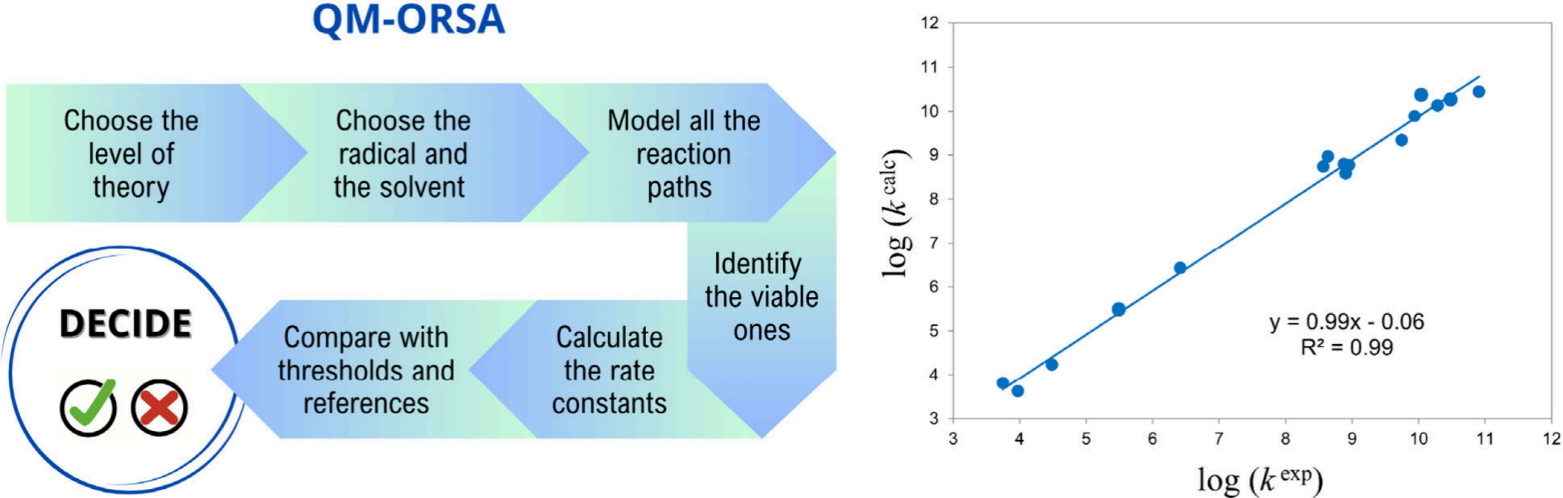
Schematic representation of the QM-ORSA protocol, and the validation of the rate constants obtained from it (*k* ^calc^) against experimental results (*k* ^exp^). Data were taken from ref 4.

Some key factors to consider when using QM-ORSA to predict AOX-I are the following:

- Level of theory: Although the density functional theory (DFT) can often produce accurate data, not all density functional approximations are reliable for kinetics. Thus, it is recommended to use one of those identified as reliable for that purpose in benchmark studies.^28–30^ Some examples are LC-xPBE, M06-2X, and BMK.
- Free radicals (FRs): Antioxidants cannot efficiently scavenge all FRs. The hydroxyl radical (^•^OH) quickly reacts with most molecules, i.e., it cannot be intercepted before damaging biological targets. On the contrary, O_2_^•–^ and H_2_O_2_ are not considered oxidants.^21,31,32^ Therefore, using any of these species in the calculations will likely generate results that do not represent biologically relevant AOX-I. Peroxyl radicals (ROO^•^), on the other hand, have been proposed as the most likely targets of antioxidants^33^ and are an appropriate choice in computational studies.
- The environment: Lipid and aqueous environments are the two relevant media to consider for AOX-I mechanisms. In aqueous solution, antioxidant acid–base equilibria influence the scavenging process since their protonated and deprotonated forms can display different reactivities. Thus, charged species should also be included in the modeling when present in significant amounts at the pH of interest (typically 7.4). In Scheme 2, 3,5-dihydroxy-4-methoxybenzyl alcohol (DHMBA) is used to illustrate the role of acid–base equilibria in radical scavenging.
- Mechanisms and reaction paths: While not all of them may be relevant, all possible reaction pathways for every possible reaction mechanism should be modeled to obtain a comprehensive rate coefficient. The kind and number of chemical routes involved in such mechanisms would depend on the antioxidant candidate. For DHMBA (Scheme 2), there are 12 possible reactions for the neutral species and 11 for the anion.
- Thermochemistry: Computed Gibbs free energies of reaction allow for the identification of exergonic reaction paths (Δ*G* < 0). For the DHMBA + HOO^•^ reaction (Scheme 2), there are five exergonic *f*-HAT reactions, three for the neutral species, and two for the anion.^34^
- Kinetics: Rate constants should be calculated for each path identified as exergonic, considering the limit imposed to them by diffusion through the solvent. Otherwise, the calculated rate constants might correspond to reactions that take place faster than the reactants encounter, which lacks physical meaning. Then the overall rate coefficient is calculated by taking the sum of the rate constants of the individual reactions weighted by the molar fraction of the reactants at the pH of interest (0.51 for neutral DHMBA, and 0.49 for its anion, at pH 7.4, Scheme 2). If the modeled radical is involved in acid–base equilibria, its molar fraction should be considered as well for the calculated results to be in line with the experiments.
- Predicting AOX-I: Comparisons are crucial to identify a molecule candidate as a viable free radical scavenger. Proteins and DNA are less reactive toward FRs than lipids. Therefore, the *k* value of the polyunsaturated fatty acid (PUFA) + HOO^•^ reaction ((1.18–3.05) × 10^3^ M^–1^ s^–1^)^35^ is a reasonable threshold to establish if a molecule can efficiently scavenge FRs before damaging these biomolecules, provided that the same radical is used for the comparison. Using a reference antioxidant, Trolox for example (*k*_HOO^•^_ = 8.96 × 10^4^ M^–1^ s^–1^, in aqueous solution, at pH 7.4),^36^ helps quantify and interpret such efficiency.

**Scheme 2:**
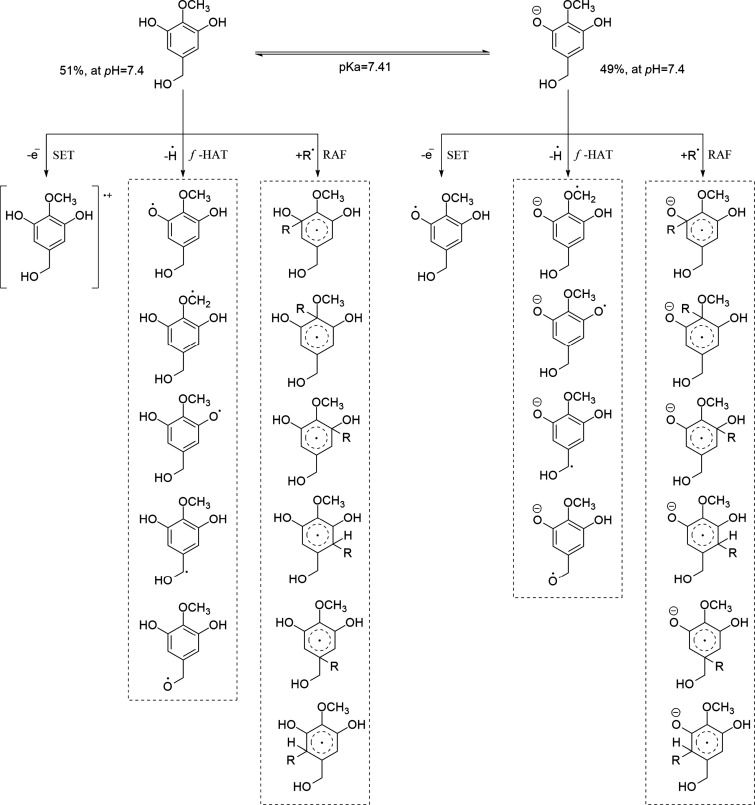
Reaction Paths Involved in the AOX-I of DHMBA, Considering the Presence of Its Predominant Forms at Biological pH

Some of the results obtained using QM-ORSA are plotted in Figure 3. Caffeine, melatonin, and uric acid are seen to be ineffective hydroperoxyl radical scavengers because the computed *k* values are lower than that of PUFA. Thus, their AOX, if any, would arise through activity other than AOX-I. On the other hand, all the molecules included in Figure 3, other than the last four, are expected to be more effective than Trolox in AOX-I.

**Figure 3:**
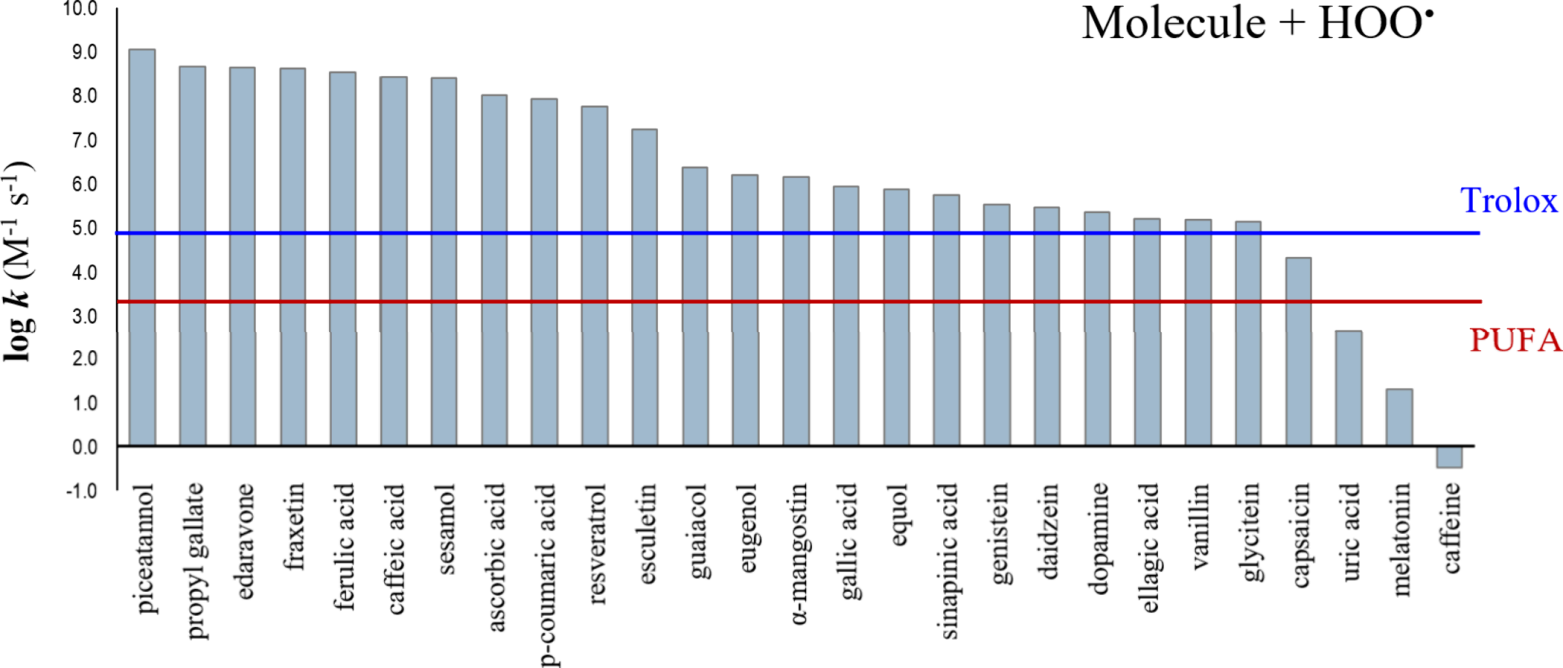
Efficiencies of different compounds as FR scavengers in aqueous solution, based on kinetics (data from ref 37). Trolox and PUFA are placed in the plot for comparison purposes.

In summary, kinetics-based approaches, such as the QM-ORSA protocol, allow for the full complexity of AOX-I to be considered. They provide a reliable framework for predicting whether a molecule will behave as a free radical scavenger, establishing trends in such activity, and obtaining results directly comparable with experiments.

## Sequestering Redox Metals (Prevention, AOX-II)

As previously mentioned, ^•^OH is so reactive that antioxidants cannot efficiently intercept it before it damages biomolecules. However, indirectly reducing the damage caused by ^•^OH may be possible by inhibiting its production.^38^ Antioxidants that behave as ^•^OH inhibiting ligands (OILs) (Figure 4) perform this function by chelating redox metals, thereby preventing their reduction and, consequently, Fenton-like reactions. More details on the role of these reactions in the ^•^OH production can be found elsewhere.^39,40^

**Figure 4:**
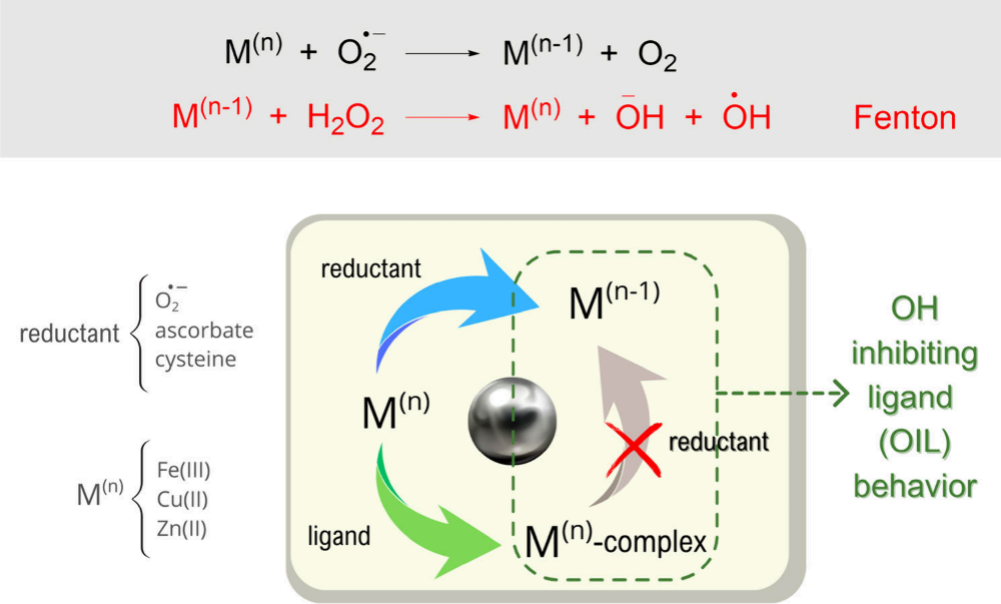
Fenton-like reactions and OIL behavior.

In our group, we use copper as a redox metal to model AOX-II. Its role in oxidative stress (OS) has been documented.^41,42^ When combined with ascorbate, copper produces ^•^OH through a complex process that initiates with the Cu­(II) reduction to Cu­(I),^43^ and its complexes mimic superoxide dismutase (SOD). To evaluate the ability of an antioxidant candidate to prevent the reduction stage by chelating Cu­(II), “free” copper is modeled coordinated to four water molecules, in a near-square-planar arrangement. This arrangement has been identified as the most likely one in aqueous solution.^44^ Even for relatively small molecules, several complexes can be envisioned, as illustrated in Scheme 3 using catechol as a model antioxidant The various complexes can be interconnected by protonation–deprotonation equilibria, which are expected to be pH-mediated, with the most abundant species being determined by the Maxwell–Boltzmann distribution (MBD). According to ref 3, only one of the Cu­(II)-catechol chelates is expected to be formed in significant amounts (marked with a box in Scheme 3) and can be produced through different routes (Figure 5). Its population, according to MDB, is more than 99%.

**Scheme 3:**
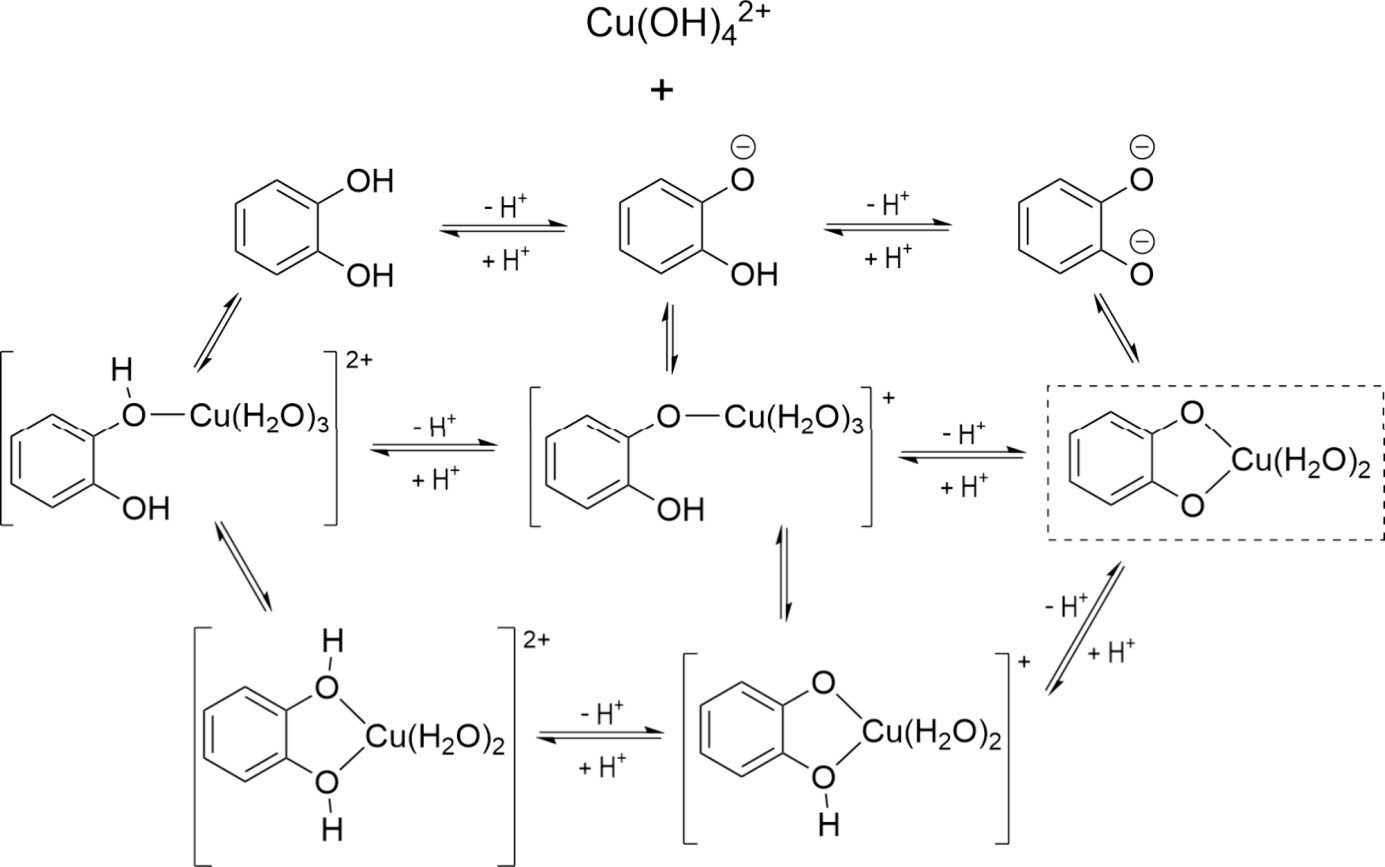
Different Cu–Catechol Complexes and their pH-Mediated Interconnection; The Most Abundant Species in Shown in the Dotted Box

**Figure 5:**
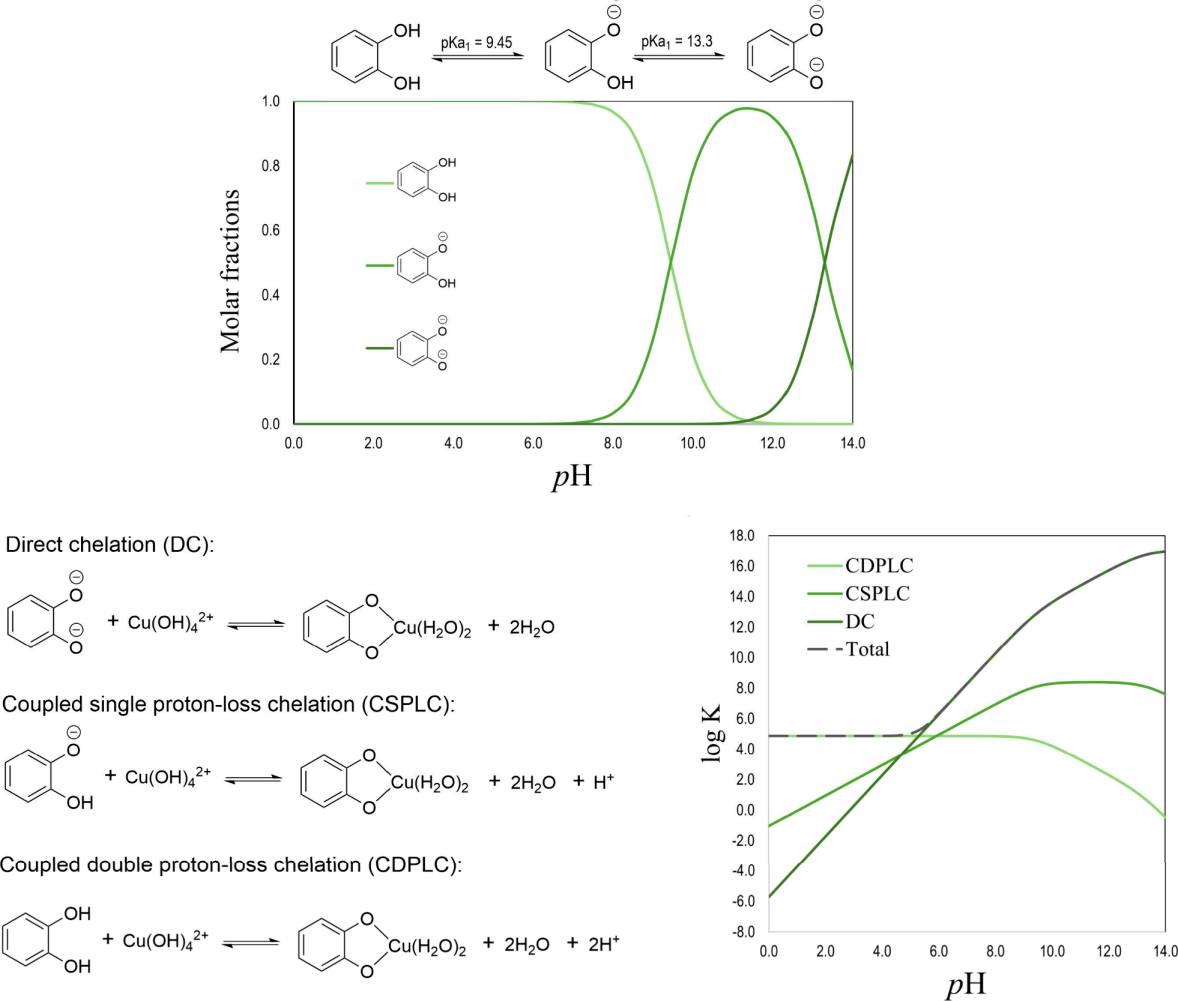
Catechol acid–base equilibria and possible routes involved in the formation of the most likely Cu–catechol complex (data taken from ref 3).

The pH modulates the contribution of each route to chelation. It also influences Cu­(II) complexes’ speciation for catechol and other ligands.^45^ In addition, pH lowering is expected when chelation involves CSPLC and CDPLC mechanisms (Figure 5), which is in line with experimental evidence.^46^ The pH effect can be included in the calculation of apparent equilibrium constants^47^ by using molar fractions and conditional Gibbs free energies (Δ*G*′).^1,3^

Once the most abundant complexes are identified, the next step is to evaluate whether they can inhibit Cu­(II) reduction and to what extent. As is the case for AOX-I, kinetics and comparisons with some appropriate references are crucial for assessing AOX-II. In this case, the strategy consists of estimating *k*(M^(*n*)^ + reductant) for the reactions involving “free” M^(*n*)^ and the M^(*n*)^ complex, the ratio between them allows for the quantification of the extent of OIL behavior. Although different reductants can be used, O_2_^•–^ and ascorbate are reasonable models since they are likely to be present in living systems and allow accounting for strong and moderate reductants. Using this approach, melatonin and its metabolites, cyclic 3-hydroxymelatonin (3OHM) and *N* ^1^-acetyl-*N* ^2^-formyl-5-methoxykynuramine (AFMK), have been proposed as capable of fully inhibiting Cu­(II) to Cu­(I) conversion, suggesting that they may be excellent for decreasing OS via AOX-II.^1^

There is another mechanism by which OIL molecules can exert protective effects even if they are unable to completely inhibit metal reduction through chelation. Since the ligands bind to the M^(*n*–1)^ species, they will be located at the ^•^OH site of formation. Thus, they may scavenge this radical *in situ*. This hypothesis has been tested and proven viable.^4,47^

## Restoring Damaged Biomolecules (Repairing, AOX-III)

The half-life of OH radicals is 10^–9^ s,^48^ which means they will not diffuse to locations remote from their formation site. They will oxidize almost any molecule in their vicinity, including lipids, proteins, and DNA (Scheme 4). That damage cannot be prevented, but antioxidants may be able to repair it. Glutathione,^2,49^ as well as melatonin and its metabolites,^50,51^ can reverse some of the lesions on the DNA structure, and restore its pristine form, and uric acid can repair tryptophanyl radicals.^52^

**Scheme 4:**
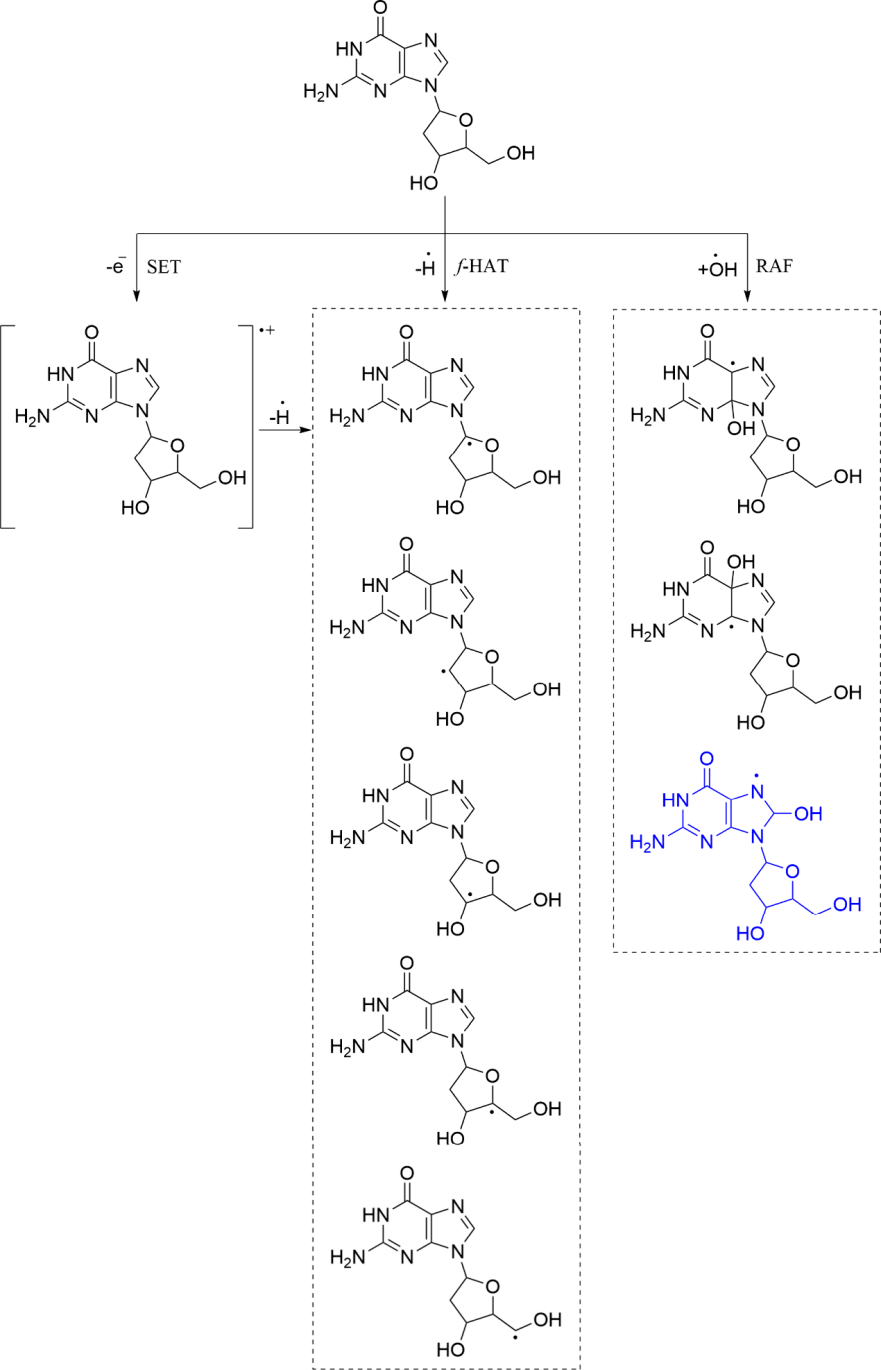
Products of 2dG Oxidation, Initiated by ^•^OH; 8-OH-dG Is Highlighted in Blue

Most biomolecules are large, and OS-induced damage involves different mechanisms and may yield multiple products. This poses a challenge in the use of computational approaches to study AOX-III. However, studies have shown that insights into AOX-III processes can be obtained using simple models of biomolecules. For example, a simplified model of linoleic acid was shown to be a good mimic for lipids.^53^ For amino acid residues, models used by Prof. Radom’s group reproduced experimental trends.^54–56^ To explore damaged DNA, 2′-deoxyguanosine (2dG) is a reasonable model as it is the nucleic base that oxidizes the easiest. Its ^•^OH-initiated oxidation starts by SET, followed by deprotonation, which yields the same products as *f*-HAT;^57^ and RAF pathways produce adducts, with the OH covalently bonded to the imidazole ring. Of particular importance is the adduct known as 8-OH-dG (highlighted in blue in Scheme 4). When further oxidized via SET, it produces 8-oxo-dG, one of the most common DNA lesions. Both 8-OH-dG and 8-oxo-dG are considered OS biomarkers.^58,59^

The reaction mechanism involved in AOX-III depends on the damage inflicted on the biomolecules. It seems logical to think that if molecules lose an electron or an H atom, they can be repaired through SET or *f*-HAT from antioxidants, respectively. Unfortunately, this reasoning might not apply, as illustrated by the repair of tryptophanyl radicals by uric acid. To reproduce the experimental data, it was required to include in the calculations two species of tryptophan (Trp_(−H)_^•^, Trp^•+^) and three species of uric acid (H_3_Ur, H_2_Ur^–^, and HUr^2–^). Considering only the most abundant species at physiological pH, Trp_(−H)_^•^ and H_2_Ur^–^, leads to a discrepancy in *k* with the experiment of 4 orders of magnitude.^52^ Moreover, it was demonstrated that the repair of Trp_(−H)_^•^ and Trp^•+^ does not involve SET or *f*-HAT mechanisms, as might be assumed. Instead, the so-called “proton–electron sequential transfer” (PEST) and “sequential proton gain electron transfer” (SPGET) combined contributions to the overall AOX-III were found to account for more than 99% of the repair process. This mechanistic proposal is supported by the good agreement of the calculated rate constants with the experimental values.

Glutathione has been identified as very efficient for restoring the lost H to C′-centered guanosyl radicals. The transfer is predicted to occur from the thiol group at diffusion-controlled rates at pH 7.4.^2^ However, under more basic conditions, the situation changes. Because of the acid–base equilibria of glutathione, the H in the thiol group is not present after its second deprotonation. The estimation of the molar fractions indicates that at pH 8.75 the apparent rate constant for the repairing process will be half of that at pH 7.4, and it will be one, two, three, and 4 orders of magnitude lower at pH values equal to 9.7, 10.7, 11.7, and 12.7, respectively. Other factors should be considered, such as competing reactions under physiological conditions, for example, reactions between O_2_ and C′-centered guanosyl radicals, which would prevent the *f*-HAT repairing route from happening.

Although there are no evident ways to revert OH-adducts formation, a plausible mechanism has been proposed that may contribute to such DNA repair (Scheme 5).^50^ It consists of a *f*-HAT from antioxidants to the radical in the imidazole ring, followed by dehydration. Since the step that determines the viability of the overall reaction is the first one, good H donors are expected to act as AOX-III species capable of repairing this kind of DNA lesion. This was tested for melatonin and its metabolites. In the case of 8-OH-dG, only 4-hydroxy- and 6-hydroxymelatonin were found to be efficient for that purpose, with rate constants in the range 10^4^–10^5^ M^–1^ s^–1^.^50^ The dehydration step does not depend on the antioxidant candidate and can occur either as an elementary reaction or through a sequential dehydroxylation-deprotonation process. While both are predicted as viable at pH 7.4, the latter depends on the environment’s acidity.

**Scheme 5:**
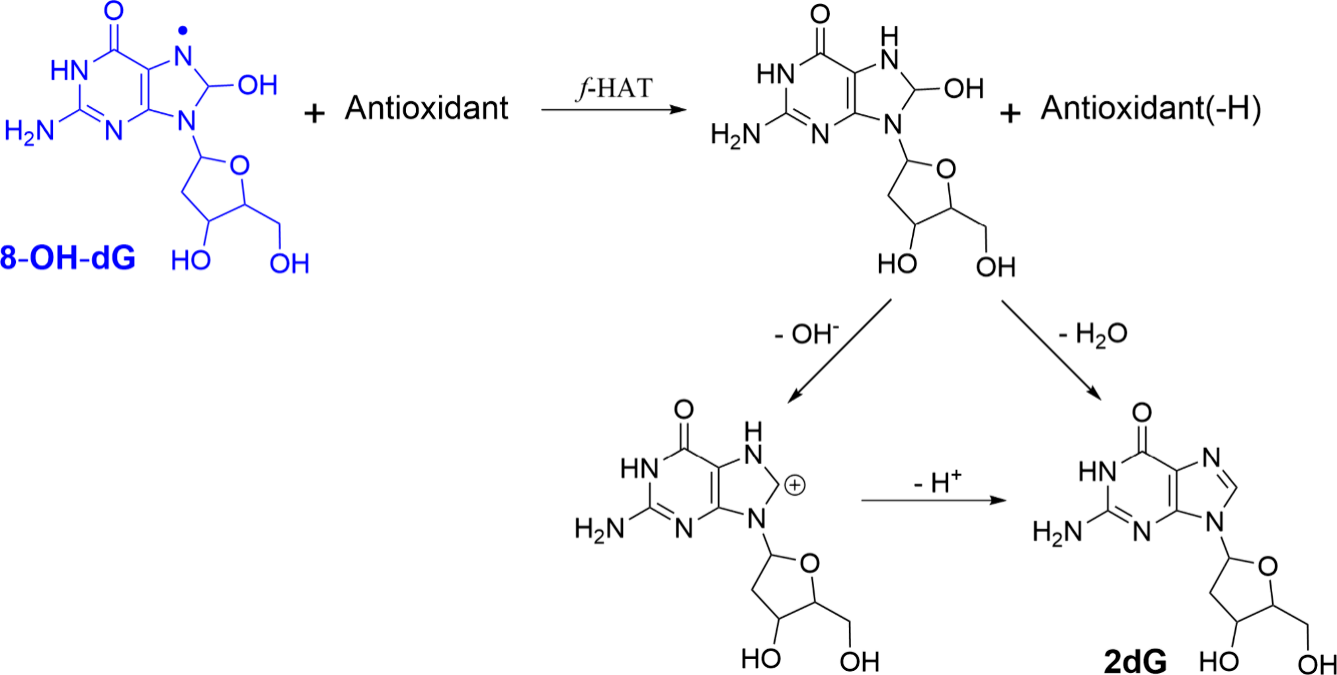
Reaction Pathway Proposed in Reference 50 for the Repair of 8-OH-dG DNA Lesions

As these case studies demonstrate, pH must be considered when modeling AOX-III, as is the case for AOX-I and AOX-II. Including the effect of pH in computational approaches is relatively easy. It requires estimating the molar fractions for the species of interest, provided that the p*K*_a_ values are known and, if not, there are reliable and simple ways to calculate them.^60–62^

## Toxic Effects

In contrast to their beneficial effects, antioxidants can cause molecular damage, and this risk is equally important to evaluate. As already mentioned, ascorbate promotes ^•^OH production in the presence of redox metals, acting as a pro-oxidant. This behavior is not exclusive to ascorbate. Other examples are curcumin, flavonoids, phenolic compounds, carotenoids, and purines.^63–66^ The potential toxicity of purines arises from the role of their anions as reducing agents. Thus, they can promote Fenton-like reactions. In this context, uric and 1-methyl uric acids pose a higher risk than other purines (Figure 6). Because of their lower p*K*_a_ values, a higher proportion of the anionic form, i.e., the active one, would be available to reduce M^(*n*)^, at pH 7.4.^65^

**Figure 6:**
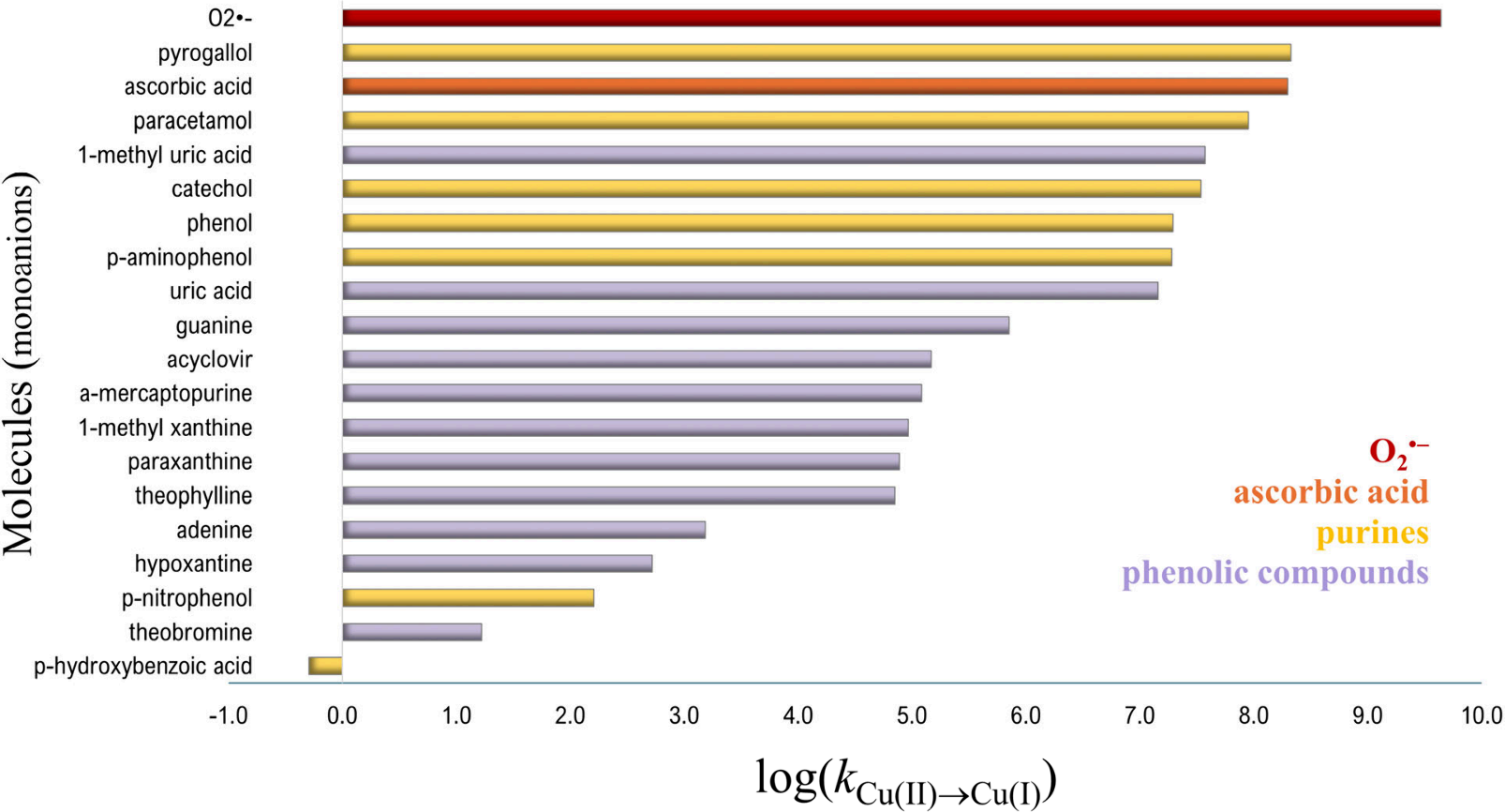
Trend of the pro-oxidant behavior of deprotonated molecules via Cu­(II) reduction (data from refs 3, 53, and 65).

Phenolates are also excellent reductants, except for those with strong electron-withdrawing groups, such as -NO_2_. They have been proven to rapidly convert Cu­(II) into Cu­(I). Among those compared in Figure 6, pyrogallate and paracetamol are the strongest pro-oxidants. The rate constants associated with their one-electron Cu­(II) reduction are similar to that of ascorbate.

The toxicity of phenols may involve other pathways. Phenoxyl radicals (^•^PhR) yielded from their AOX-I activity, through *f*-HAT or SPLET (Scheme 1), can still be hazardous to biomolecules by potentially propagating chain reactions. While ^•^PhR do not pose a risk to DNA integrity, most of these radicals can abstract an allylic H from PUFAs, initiating lipid peroxidation processes.^3^ It is worth mentioning that the better H donor a compound is, the lower the ability of its radical to damage other molecules.

Regarding *f*-HAT from proteins to ^•^PhR, the viability of such processes is modulated by the amino acid residue, with cysteine being the most vulnerable. However, the most common damage that can be inflicted on proteins is benzoquinone imine (BQI)-induced arylation. Paracetamol can be considered a paradigmatic example of this deleterious effect.^3^ BQIs are produced from its ^•^PhR (Figure 7). They then covalently bind to deprotonated thiol groups in amino acid residues, causing arylation, which has been held responsible for the paracetamol hepatotoxicity.^67,68^ Fortunately, there are promising compounds for preventing this kind of damage, including glutathione.^69^

**Figure 7:**
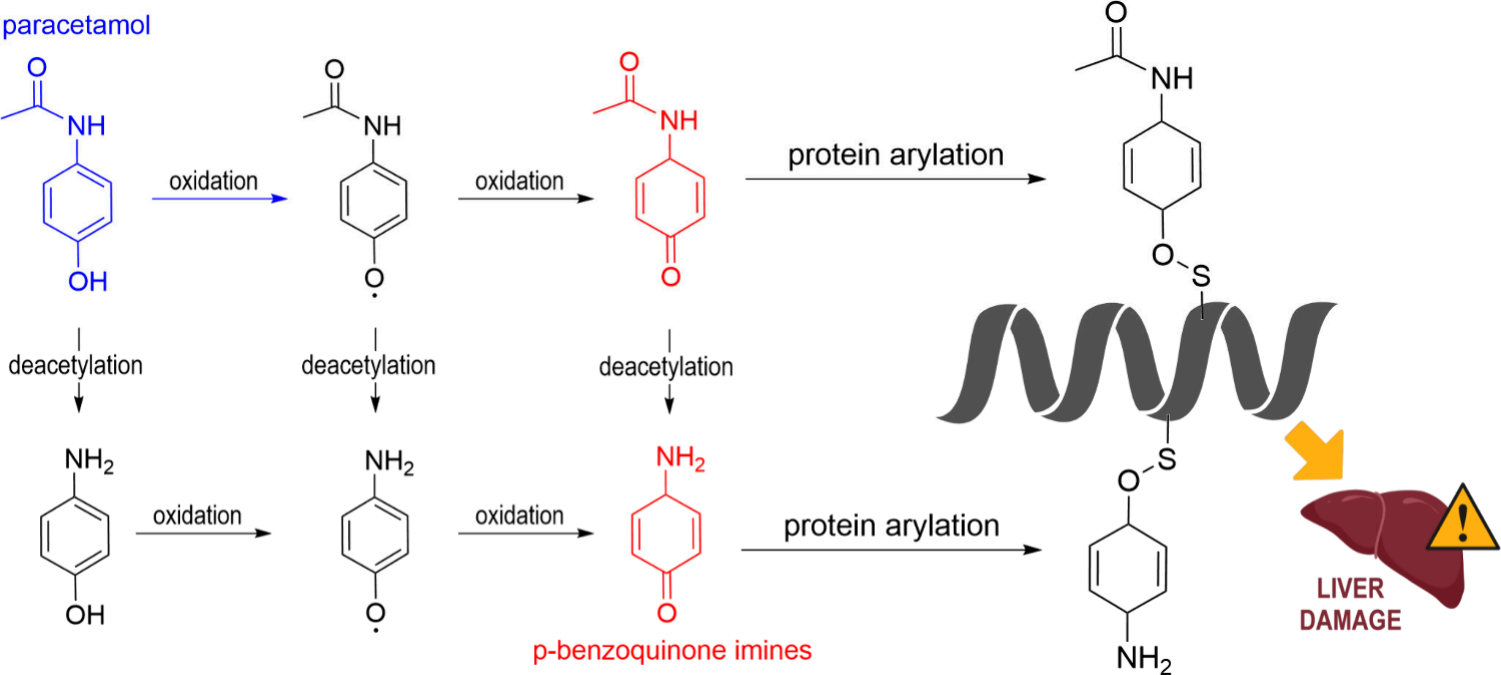
Illustration of paracetamol oxidation, leading to protein arylation and ultimately to liver damage.

## Designing New Antioxidants

The title of this section might raise the following question: With so many antioxidants of natural origin, why keep searching for new ones? The answer can be twofold:

1. Toxicity and versatility: It has been previously stated that “most phenolic compounds have pro-oxidant activity at low concentrations, unlike synthetic antioxidants”,^70^ and also that “more efforts should be directed toward the development of synthetic compounds, able to overcome the above-stated problems but endowed with the versatility of their natural counterparts”.^71^ Thus, designing new antioxidants with lower toxicity than the natural ones, without losing versatility, is an appealing approach to counteract OS. A versatile antioxidant (AOX-I + AOX-II + AOX-III) is expected to be more efficient against OS than a single-purpose one (just AOX-I, for example), and if it can exert other health benefits even better.
2. Multifunctionality: Many of the most serious health disorders that humankind faces today are multifactorial and OS-related, including Alzheimer’s^72^ and Parkinson’s^73^ diseases. Thus, developing multifunctional molecules as potential candidates to treat these diseases has become a new paradigm in drug design.^74^ For example, inhibitors of acetylcholinesterase (AChE) are commonly used to treat Alzheimer’s disease,^75^ while inhibitors of catechol-O-methyltransferase (COMT)^76^ and monoamine oxidase-B (MAO-B)^77^ are used as neuroprotectors in Parkinson’s disease. Thus, molecules capable of inhibiting these enzymes and simultaneously behaving as antioxidants might be promising candidates to enhance the benefits of the current therapies.

One valid concern related to this topic is the time and cost of the research. Computational tools can significantly reduce them, provided that they are reliable enough and capture the complex chemical behavior of antioxidants, including benefits and risks. The CADMA-Chem protocol^5^ can be used for that purpose. Its primary goal is to design multifunctional molecules as potential candidates for treating OS-related diseases. It encompasses all the aspects discussed in the previous sections of this Account (Figure 8) and other factors that matter when a new molecule is intended for human or animal consumption. The designed candidates aim to be versatile antioxidants, exhibiting low toxicity and oral drug-like properties, being synthetically accessible, and acting as agonists or antagonists of the enzymatic targets associated with the disease of interest.

**Figure 8:**
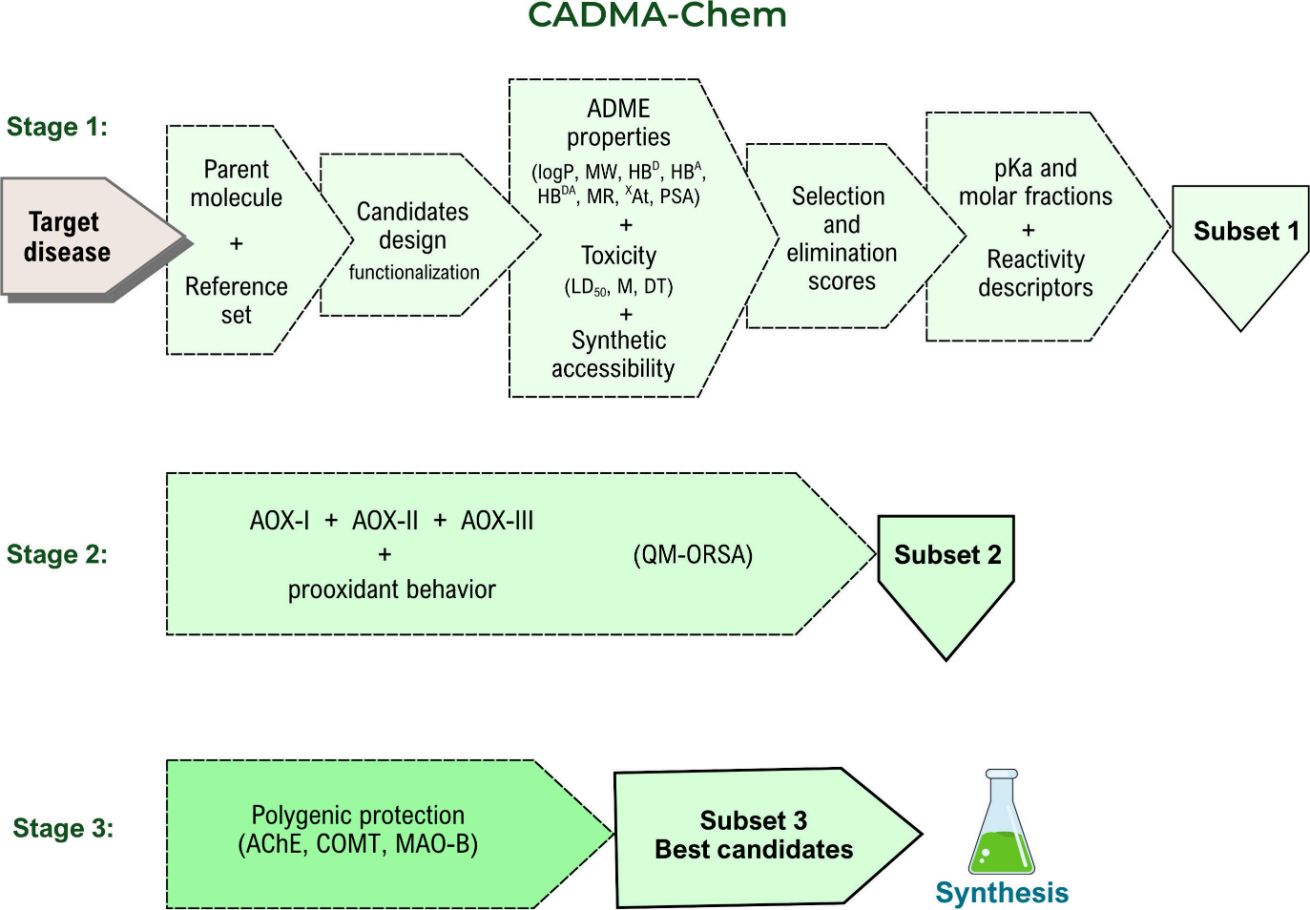
Schematic representation of the CADMA-Chem protocol, meant to find multifunctional molecules against OS. logP = octanol/water partition coefficient. MW = molecular weight; HB^D^ = number of H-bond donors; HB^A^ = number of H-bond acceptors; HB^DA^ = HB^D^ + HB^A^; MR = molar refractivity; ^X^At = number of non-H atoms; PSA = polar surface area; LD_50_ = oral rat 50% lethal dose; M = Ames’ mutagenesis; DT = development toxicity; AChE = acetylcholinesterase; COMT = catechol-O-methyltransferase; MAO-B= monoamine oxidase-B.

When using the CADMA-Chem protocol, the target might also be directly OS, instead of a specific disease. In this scenario the parent molecule might be a single-purpose antioxidant or a versatile antioxidant with relatively high toxicity. In any case, more efficient antioxidants can be developed from improvable molecular frameworks. For example, melatonin is a good antioxidant,^78^ despite its modest performance as a free radical scavenger.^79^ Thus, by slightly modifying its structure, i.e., adding functional groups, it is possible to find some derivatives with better AOX-I, without losing the other functions of the parent molecule. Some newly designed melatonin derivatives have the desired drug-like properties, considering ADME, toxicity, and synthetic accessibility;^80^ are excellent free radical scavengers with no pro-oxidant behavior;^81^ act as OIL agents;^82^ repair oxidized biomolecules;^50^ and have neuroprotective effects through inhibition of COMT, MAO-B, and AChE.^5,83,84^

Other molecules derived from natural antioxidants (chalcones,^85^ quinolines,^86,87^ and ferulic^88^ and caffeic^89^ acids) were designed using the CADMA-Chem protocol. They were identified as potential multifunctional antioxidants with low toxicity and inhibitory effects on AChE, COMT and MAO-B. Although further studies, both computational and experimental, are still required to confirm the role of these molecules as versatile antioxidants with neuroprotective effects, they are promising candidates as therapeutic agents in the treatment of Parkinson’s and Alzheimer’s. Using the same protocol, the role of protocatechuic aldehyde as a versatile antioxidant was supported (through AOX-I, AOX-II and AOX-III protection, and by inhibiting the pro-oxidant enzyme xanthine oxidase);^90^ and bergaptol derivatives were proposed as AOX-I + AOX-II + AOX-III antioxidants and selective CYP3A4 inhibitors.^91^ Some aspects of the CADMA-Chem protocol were applied to the evaluation of *N*-phenoxyethylisatin hydrazones.^92^ They were proposed as candidates for treating hyperglycemia based on their inhibition of α-glucosidase, low toxicity, and good bioavailability.

OS is involved in numerous diseases, especially in those considered multifactorial which are among the hardest to treat. Thus, the search for multifunctional antioxidants, specifically designed to treat them, might help to accelerate the discovery of therapeutic drugs that help people affected by those diseases.

## Concluding Remarks

Antioxidants are supposed to be nontoxic versatile molecules that may come from natural sources or from rational designs. They are expected to counteract the harmful effects of OS without posing other risks to human health. Such beneficial action may involve chemical routes as well as enzyme-related pathways, which can be investigated using both experimental and computational techniques, although combined approaches are the best way to address their activity. The antioxidants’ chemical protection mechanisms include: (i) Free radical scavenging reactions, also referred to as interception or AOX-I; (ii) sequestering redox metal and inhibiting their reduction, thus consequently inhibiting OH radical production via Fenton-like reaction (prevention, AOX-II, or OIL behavior); (iii) restoring oxidatively damaged biomolecules to their pristine forms (repairing or AOX-III). On the contrary, antioxidant candidates may also pose risks to human health. The products formed after their reactions with free radicals (FR) can still damage biomolecules, particularly PUFAs, and contribute to propagate FR-chain reactions; while some phenols may form benzoquinone imines and induce protein arylation. Thus, predicting a molecule as an effective and safe antioxidant requires taking all these aspects into consideration. When antioxidant activity is approached using computational tools, it is also crucial to include environmental aspects into the modeling, evaluate ADME properties, and use kinetics-based protocols that allow direct comparisons with experimental results.

A comprehensive investigation of the complex chemistry of antioxidants is expected to contribute to gaining a better understanding of their potential effects in living organisms. Important implications to human health depend on assessing their risks and benefits. Future prospects related to this area of research may include: (i) The exploration of the interactions between potential antioxidants with the enzymatic redox system. There is still little information about the effects of exogenous antioxidants on this system. Can they inhibit pro-oxidant enzymes? Can they up-regulate antioxidant enzymes? Can they modulate redox transcriptional factors? Can they have undesirable effects when they interact with the associated enzymes? (ii) The chemical fate, including toxicity, of the products formed from antioxidants through chemical and metabolic routes. Are such products beneficial or harmful? (iii) The design of versatile and safe antioxidants that might also be used in the treatment of OS-related diseases. Most of these diseases are multifactorial, and the medications currently used to treat them are far from being considered a cure. Might new, multifunctional, antioxidants contribute to enhance the efficiency of related therapies? (iv) The inclusion of emerging computational tools into the investigation of antioxidant. Can machine learning and artificial intelligence provide faster, but still reliable, predictions regarding the complex chemistry of antioxidants?

Despite the many efforts devoted so far to investigating antioxidants, there are still many aspects of their chemistry to reveal. Hopefully this account may inspire further research into this area of knowledge. One thing is certain, we will continue learning about antioxidants and how to use them to improve human health.
